# Both HDAC5 and HDAC6 are required for the proliferation and metastasis of melanoma cells

**DOI:** 10.1186/s12967-015-0753-0

**Published:** 2016-01-08

**Authors:** Jiaqi Liu, Jianying Gu, Zihao Feng, Yanhong Yang, Ningwen Zhu, Weiyue Lu, Fazhi Qi

**Affiliations:** Department of Plastic Surgery, Zhongshan Hospital, Fudan University, 180 Fenglin Road, Shanghai, 200032 China; Division of Anti-tumor Pharmacology, State Key Laboratory of Drug Research, Shanghai Institute of Materia Medica, Chinese Academy of Sciences, 555 Zuchongzhi Rd, Shanghai, 201203 China; Huashan Hospital, Fudan University, 12 Middle Urumqi Rd, Shanghai, 200040 China; Department of Pharmaceutics, School of Pharmacy, Fudan University, 826 Zhangheng Rd, Shanghai, 201203 China

**Keywords:** Melanoma, HDAC inhibitors, HDAC5, HDAC6, Proliferation, Metastasis

## Abstract

**Background:**

Histone deacetylase (HDAC) inhibitors are widely used in clinical investigation as novel drug targets. For example, panobinostat and vorinostat have been used to treat patients with melanoma. However, HDAC inhibitors are small-molecule compounds without a specific target, and their mechanism of action is unclear. Therefore, it is necessary to investigate which HDACs are required for the proliferation and metastasis of melanoma cells.

**Methods:**

We used overexpression and knocking down lentivirus to clarify the influence of HDAC5 and HDAC6 in melanoma development. Also, we introduced stable HDAC5 or HDAC6 knockdown cells into null mice and found that the knockdown cells were unable to form solid tumors. Finally, we tested HDAC5 and HDAC6 expression and sub-location in clinical melanoma tissues and tumor adjacent tissues.

**Results:**

In this study, and found that HDAC5 and HDAC6 were highly expressed in melanoma cells but exhibited low expression levels in normal skin cells. Furthermore, we knocked down HDAC5 or HDAC6 in A375 cells and demonstrated that both HDAC5 and HDAC6 contributed to the proliferation and metastasis of melanoma cells.

**Conclusions:**

This study demonstrated both HDAC5 and HDAC6 were required for melanoma cell proliferation and metastasis through different signaling pathways.

**Electronic supplementary material:**

The online version of this article (doi:10.1186/s12967-015-0753-0) contains supplementary material, which is available to authorized users.

## Background

In recent years, malignant melanoma has been reported to be one of the highest incidences among all cancers, and melanoma-related deaths increase each year. Typically, the malignant melanoma has the following characteristics: high metastasis, rapid diseases progression, poor prognosis, and high mortality. Thus, it is urgent to develop efficient drugs applied for melanoma treatment [1–3].

Some agents have emerged as inhibitors of histone deacetylases (HDACs), with consequences of chromosome remodeling, cell cycle arrest and selective toxicity to melanoma cells comparing with normal melanocytes. For example, Peng et al. [4] showed that the HDAC inhibitor sodium butyrate inhibits baculovirus-mediated transgene expression in Sf9 cells. Kuwajima et al. also found that the HDAC inhibitor butyrate inhibits the invasion of melanoma cell in Matrigel. Interestingly, Munshi et al. reported the ability of multi-HDAC inhibitors, including sodium butyrate (NaB), phenyl butyrate, tributyrin, and trichostatin A, to radiosensitize two human melanoma cell lines (A375 and MeWo) using clonogenic cell survival assays. Otherwise, NaB induced hyperacetylation of histone H4 in the two melanoma cell lines and in normal human fibroblasts [5, 6].

In 1986, Beppu and colleagues found that the antibiotic trichostatin A inhibited the growth of SV40-transformed cells in mice [7], one of the first examples of selective growth inhibition by a HDAC inhibitor.

Two compounds, vorinostat and romidepsin, have been approved by the FDA to treat refractory cutaneous T cell lymphoma [8–10]. Except these two FDA-approved agents, much more HDAC inhibitors would be tested in clinical, such as panobinostat (LBH589), givinostat (ITF2357), mocetinostat (MGCD01030), belinostat (PXD101), pracinostat (SB939), and entinostat (MS275) [11, 12]. In most reported trials, the HDAC inhibitors could be applied in combination with standard doses of other drugs, with synergistic clinical activity and without additional toxicity, suggesting a promising role of HDAC inhibitors in cancer combination therapy [13]. However, the molecular mechanism may vary with cell lines and HDAC inhibitor classes. Success in the clinic may require combination with agents that synergize with the cell cycle blocking and pro-apoptotic action of HDAC inhibitors.

The opportunity to understand and exploit a novel, nontoxic approach to cancer chemotherapy has stimulated a major effort to explore the relevant cell signaling pathways and to develop new inhibitors to HDACs. Currently, epigenetic drugs studies are relatively hot. Recently, a second generation of reportedly available HDACis have been tested in the clinic including the class I—specific agents CHR-3966 [14], chidamide (CS055/HBI-8000) [15], class I— and class II—specific AR-42 [16], and hydroxamides quisinostat (JNJ-26481585) [17] and abexinostat (PCI-24781) [18]. However, HDAC inhibitors seem to be not specific to a single HDAC, but a HDAC family. Furthermore, the inhibition of more than one HDAC may complicate the results because the HDACs have a variety of substrates. Thus, the application of non-specific HDAC inhibitors as clinical drugs may pose a potential risk.

HDAC5 protein has wide substrates and belongs to the class II HDAC alpha family. Two transcript variants encoding two different isoforms have been found for this gene. HDAC5 possesses HDAC activity and represses transcription when tethered to a promoter. HDAC5 co-immunoprecipitates with HDAC3, HDAC4 and may form multi-complex proteins [19, 20]. HDAC5 also interacts with myocyte enhancer factor-2 (MEF2) proteins [21], resulting in repression of MEF2-dependent genes [22]. Furthermore, AMP-activated protein kinase regulation of the glucose transporter GLUT4 occurs via phosphorylation of HDAC5 [23]. HDAC5 is involved in memory consolidation and targeting HDAC5 has been suggested to be avoided for the development of more selective HDAC inhibitors to treat Alzheimer’s disease [24].

By contrast, HDAC6 contains an internal duplication of two catalytic domains that appear to function independently of each other. This protein possesses HDAC activity and represses transcription. HDAC6 is involved in cell motility and catalyzes α-tubulin deacetylation [25–27], and thus, the enzyme also promotes cancer cell metastasis [28]. HDAC6 also affects transcription and translation by regulating the heat-shock protein 90 (Hsp90) and stress granules (SGs), respectively [29]. Furthermore, HDAC6 also binds to ubiquitinated proteins with high affinity [30, 31]. HDAC6 is also required for the formation of SG proteins and is instrumental in SG formation; pharmacological inhibition or genetic removal of HDAC6 abolishes SG formation.

In this present study, we showed that knockdown of HDAC5 or HDAC6 prevented proliferation and induced apoptosis of the melanoma cells. Also, we tried to link HDAC5 and HDAC6 to multiple signaling pathways. However, we have not furthermore detailed evidence to identify HDAC5 and HDAC6 how to influence cell proliferation and metastasis. Our data gave some hints that histone de-acetyltransferases could have very complicated substrate network which need us to make efforts to discover.

## Methods

### Cell lines and materials

The human melanoma cell lines A375 and A2058 used in this study were obtained and grown as previously described [32]. The following reagents and primary antibodies were used: HDAC1, rabbit, Santa Cruz SC-7872; HDAC2, mouse, Santa Cruz SC-9959; HDAC3, rabbit active motif 40968 IB; HDAC4, rabbit, active motif 40969; HDAC5, mouse, Santa Cruz SC-133225; HDAC6, rabbit, Santa Cruz SC-11420; HDAC7, mouse, Santa Cruz SC-74563; HDAC8, mouse, Santa Cruz SC-17778; anti-HDAC9 antibody (ab18970), Abcam; anti-HDAC10 antibody (ab18971), Abcam; anti-HDAC11, ab18973, abcam; anti-ERK (tERK), anti-phospho-ERK (pERK), anti-AKT (tAKT), anti-phospho-AKT-T308 (pAKT-T308), anti-phospho-AKT-S473 (pAKT-S473), anti-caspase 3, anti-cleaved caspase 3, anti-caspase 8, anti-cleaved caspase 8, anticaspase 9, anti-cleaved caspase 9 (Cell Signaling Technology, Danvers, MA, USA), anti-EGFR (tEGFR), anti-Cathepsin-D, anti-VEGF, anti-MMP9, anti-E-cadherin, and anti-GAPDH (Santa Cruz Biotechnology Inc., Santa Cruz, CA, USA). Lipofectamine 2000 reagent was obtained from Invitrogen (Carlsbad, CA, USA, Cat. No 11668-019).

### RNA extraction

RNA from the melanoma cell lines and normal skin cell line was extracted using the TRIzol^®^ Reagent (Invitrogen) as indicated by the manufacturer’s instructions. To avoid DNA contamination, RNase-free DNase I was used. The RNA concentration and quantity were assessed by absorbance at 260 nm using a DNA/Protein Analyzer (NanoDrop; Invitrogen).

### Semi-quantitative and real-time RT-PCR

Real time PCR was performed in a 20 μl reaction system with a total of 2 μg of RNA (M-MLV Reverse Transcriptase, TOYOBO CO., LTD. Life Science Department OSAKA JAPAN). Quantitative RT-PCR and real-time RT-PCR were performed with an ABI PCR Thermal Cycler Dice Detection System and SYBR green dye (TOYOBO CO., LTD. Life Science Department OSAKA JAPAN) according to the manufacturer’s recommended protocol. The following primers were used: HDAC1, forward primer CTACTACGACGGGGATGTTGG, reverse primer GAGTCATGCGGATTCGGTGAG; HDAC2, forward primer ATGGCGTACAGTCAAGGAGG, reverse primer TGCGGATTCTATGAGGCTTCA; HDAC3, forward primer CCTGGCATTGACCCATAGCC, reverse primer CTCTTGGTGAAGCCTTGCATA; HDAC4, forward primer GGCCCACCGGAATCTGAAC, reverse primer GAACTCTGGTCAAGGGAACTG; HDAC5, forward primer TCTTGTCGAAGTCAAAGGAGC, reverse primer GAGGGGAACTCTGGTCCAAAG; HDAC6, forward primer AAGAAGACCTAATCGTGGGACT, reverse primer GCTGTGAACCAACATCAGCTC; HDAC7, forward primer GGCGGCCCTAGAAAGAACAG, reverse primer CTTGGGCTTATAGCGCAGCTT; HDAC8, forward primer TCGCTGGTCCCGGTTTATATC, reverse primer TACTGGCCCGTTTGGGGAT; HDAC9, forward primer AGTAGAGAGGCATCGCAGAGA, reverse primer GGAGTGTCTTTCGTTGCTGAT; HDAC10, forward primer CAGTTCGACGCCATCTACTTC, reverse primer CAAGCCCATTTTGCACAGCTC; HDAC11, forward primer ACCCAGACAGGAGGAACCATA, reverse primer TGATGTCCGCATAGGCACAG; CDKN1A, forward primer ACATCGCCAAGGAAAAACGC, reverse primer GTCTGTTTCGGTACTGTCATCC; and GAPDH, forward primer ACAACTTTGGTATCGTGGAAGG, reverse primer GCCATCACGCCACAGTTTC.

### Designing shRNA sequences of hHDAC5 or hHDAC6, Lentivirus packaging and stable cell line construction

We used online sofaware to design the shRNA sequences of hHDAC5 and hHDAC6: http://rnaidesigner.lifetechnologies.com/rnaiexpress/setOption.do?designOption=shrna&pid=-1447534201472129460. The relative knockdown efficiency was tested by transiently transfecting into HEK-293 cells. The shRNA sequences were listed as follows:

For knocking down hHDAC5: 851, GCAAGGATGGGACTGTTATTA; 860, GGACTGTTATTAGCACCTTTA; 1243, GGCAAGTTCATGAGCACATCC. For knocking down hHDAC6: 2018, GCTATGATCATGGCACCTTC T; 2338, GGTGGCTATAACCTGACATCC; 2511, GAAGGTAGAAGACAGAGAAGG.

After measuring the best shRNA seq to knock down hHDAC5 or hHDAC6, then package the lentivirus plasmids containing the shRNA seq with VSVG and delta8.9 package plasmids to form lentivirus particle.

Amplification of lentivirus was performed via standard methods in sub-confluent HEK293 cells. Infection of melanoma cell lines was performed in the presence of polybrene (Sigma) at a final concentration of 8 µg/ml. The cells were incubated with the lentivirus mixture for 72 h, digested with trypsin to fresh growth medium, and then sorted with green fluorescence for stable expression or knockdown. Constructed stable cell lines were amplified and saved for future experiments.

### Colony formation

For the soft agar colony formation assay, pre-treated melanoma cells that stably knocked down HDAC5 or HDAC6 were grown on a plate containing 1 % base agar and 0.5 % top agar. After approximately 3–4 weeks of incubation, the colonies were counted with a dissecting microscope. All experiments were independently repeated at least three times.

### Cell proliferation

The inhibition effects of HDAC5 or HDAC6 were evaluated by cell counting kit-8 (CCK-8; Dojindo Molecular Technologies Inc., Gaithersburg, MD, USA). The cells were passaged in a 48-well plate with RIPM1640 and 10 % FBS for 1–7 days. The IC50 value was calculated using Statistical Package for the Social Sciences (SPSS) software version 12 (SPSS Inc., Chicago, IL, USA).

### Cell invasion assay

Cell transwell assays were performed using FalconTM Cell Culture Inserts (BD353097,BD company, USA, New Jersey) according to the manufacturer’s instructions. After 24 h of incubation, the remaining cells in the upper chamber were removed with cotton swabs. The cells on the lower surface of the membrane were fixed in 4 % paraformaldehyde and stained with 0.5 % crystal violet. Cells in at least three random microscopic fields (magnification ×10) were counted and photographed. All experiments were performed in duplicate and repeated three times.

### Flow cytometry with annexin V-FITC and PI staining

The melanoma cells were divided into three groups: (1) scramble (scr) melanoma cells as a negative control; (2) stably knockdown HDAC5 melanoma cells; and (3) stably knockdown HDAC6 melanoma cells. The melanoma cells were collected, washed twice with PBS, and then stained with propidium iodide and annexin V. Cell cycle and apoptotic analyses were performed by flow cytometry (FCM) as previously described using a FACS Calibur system [33]. Apoptotic cells were analyzed by quadrant statistics of the propidium iodide negative and annexin V-positive cells.

### Western blotting

Cells were washed twice in PBS; Then, the whole cell lysates were collected by adding RIPA lysis for 20 min, centrifuging for 15 min at 13000 rpm and obtaining the supernatant. Densitometry analysis was performed with Quantity One software (Bio-Rad, Hercules, CA, USA). The relative expression level of each protein was normalized by dividing the level of target protein by the level of GAPDH for each sample.

### Melanoma samples and immunohistochemistry

Melanoma samples were acquired from Zhongshan Hospital, Fudan University. A physician obtained informed consent from the patients. Immunohistochemistry (IHC) was performed as described [34]. To quantify the IHC result of positive staining, the tissue areas of five ductsim each sample were microscopically examined and analyzed by an experienced pathologist. Images were captured using a charge-coupled device camera and analyzed using Motic Images Advanced software (version 3.2, Motic China Group). Average of staining score was calculated by dividing the positive areas with total areas. Data obtained were expressed as mean values ± SD. Differences were considered significant if the p value was less than 0.001.

### In vivo tumor xenograft study

Five to seven week old female BALB/c-nu/nu nude mice were purchased from the Shanghai Institute of Materia Medica, Chinese Academy of Sciences (Shanghai, China). Tumors were initiated by injecting 2 x 10^6^ cells into the armpit of the nude mice. The mice were randomized and assigned to the control or experimental groups.

Mice in the control group were administered 0.1 ml of RPMI1640. The tumors were measured every 5 days with microcalipers, and tumor volume was measured using the formula π/6 larger diameter (smaller diameter)^2^ [35].

### Statistical analysis

Statistical analysis was performed using the Statistical Package for the Social Sciences (SPSS) software version 12 for Windows (SPSS Inc., Chicago, IL, USA). Student t-tests were used to determine the statistical significance of the differences between the experimental groups. A P-value of <0.05 was considered significant. Graphs were created using Microcal Origin software (version 3.78; Microcal Software, Inc., Northampton, MA, USA).

## Results

### HDAC5 and HDAC6 are overexpressed in melanoma cells

For effective prevention of melanoma development using HDAC inhibitors, further study of the exact mechanisms for inhibition of HDACs is very important. To assess the high expression of HDACs in melanoma cells comparing with normal skin cells, we tested the expression of all HDACs at the protein and mRNA levels.

**Fig. 1:**
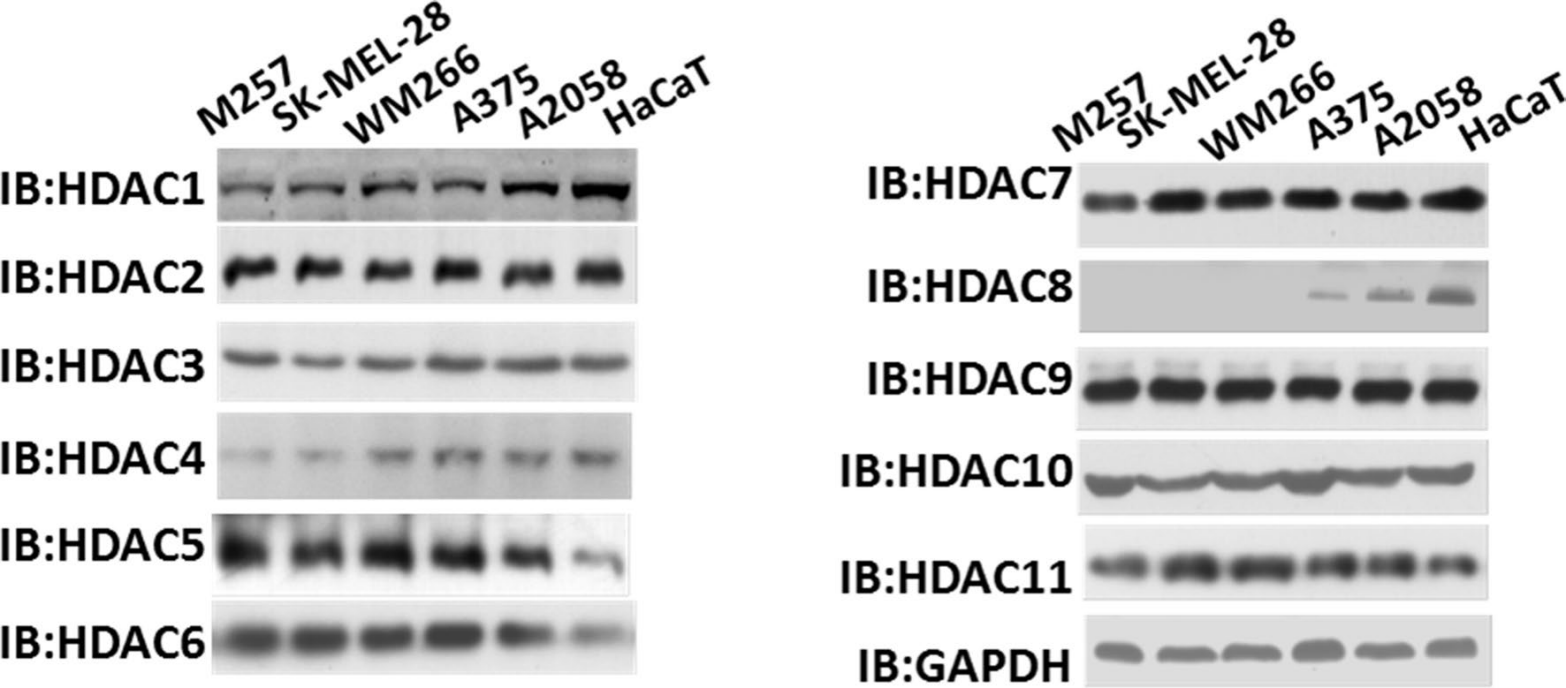
HDAC5 and HDAC6 were overexpressed in melanoma cells. Western blots were used to identify all HDACs except sirtuins in M257 cells, SK-MEL-28 cells, WM266 cells, A375 cells, A2058 cells and HaCaT normal skin cells. The antibodies used in this figure are listed in “Methods”. GAPDH was used as an internal control

As shown in Fig. 1 and Additional file 1: Figure S1, when tested for expression levels, both HDAC5 and HDAC6 had higher protein levels in melanoma cells (M257, SK-MEL-28, A375 and A2058 cells) than normal skin cells (HaCaT). Consistent with protein levelmRNA expression of HDAC5s, the mRNA expression of HDAC5 and HDAC6 in A375 and A2058 cells was 30 ± 3 times and 78 ± 19 times, respectively compared to in HaCaT cells. However, HDAC8 had nearly no expression in melanoma cells but was expressed in normal skin tissue. Similar results were observed for HDAC1 and HDAC3, suggesting that some HDACs may function as melanoma suppressors.

### Stable knockdown of HDAC5 and HDAC6 in A375 cells

To clarify the roles of HDAC5 and HDAC6 in melanoma progression, we designed three shRNA sequences for knocking down HDAC5 or HDAC6. We selected the most efficient shRNA sequences of knocking down HDAC5 or HDAC6, as shown in Additional file 2: Figure S2a and b. Furthermore, we packed the lentivirus and infected the A375 cells to construct cell lines that stably knocked down HDAC5 or HDAC6.

**Fig. 2:**
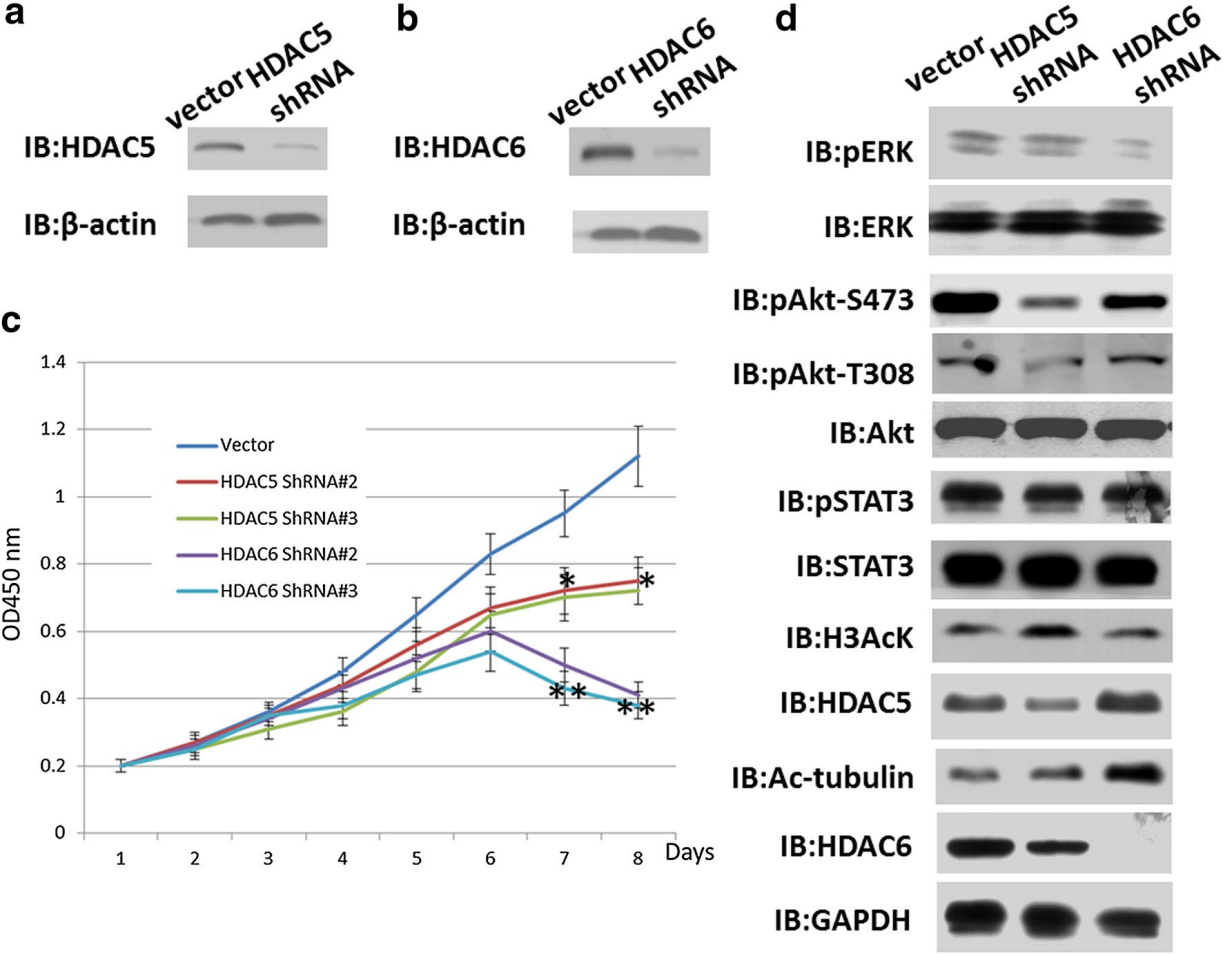
Knockdown of HDAC5 or HDAC6 inhibited the proliferation of A375 cells. The stable cell line of A375 cells with HDAC5 or HDAC6 knockdown were constructed using shRNA primers. **a** and **b** Western blotting was used to detect HDAC5 or HDAC6 expression in A375 cells. β-actin was used as an internal control. **c** CCK8 was used to count the cell number of stably knocked down HDAC5 or HDAC6 in A375 cells. Cell viability was measured using the Cell Counting Kit-8 (CCK-8, Dojindo Laboratories, Kumamoto, Japan) according to the manufacturer’s instructions. Transiently transfected cells were seeded in a 96-well plate and then cultured at 24-hour intervals for 5–7 days. Cell viability was then measured using the CCK-8 assay. Absorbance was measured at 450 nm as an indicator of cell viability. All experiments were independently repeated at least three times. *p value <0.01, **p value <0.001. p value <0.05 was considered as significant differences. **d** Western blotting was used to detect the signaling pathway related to proliferation. Acetylated-Histone H3 and acetylated-a-tubulin were used as control for monitoring HDAC5 and HDAC6 knocking down results, respectively. The antibodies used for western blotting are listed in Methods

We used CCK8 to identify cell proliferation after reducing HDAC5 or HDAC6 expression levels in melanoma cells. As shown in Fig. 2c, inhibiting HDAC5 or HDAC6 prevented cell proliferation, especially HDAC6 stably knockdown in A375 cells induced significant arrest of cell growth comparing to scramble A375 cells(**p < 0.001) while HDAC5 stably knockdown in A375 cells has also significant decrease in growth rate (*p < 0.01). Interestingly, HDAC5 and HDAC6 may influence melanoma cell proliferation through different pathways. Therefore, in the next step, we assessed the multiple pathways involved in melanoma cell growth and proliferation. We found that knocking down HDAC5 presented lower Akt phosphorylation, whereas knocking down HDAC6 dramatically decreased the phosphorylation of ERK (Fig. 2c).

### Both HDAC5 and HDAC6 reduce apoptosis and promote metastasis of melanoma cells

**Fig. 3:**
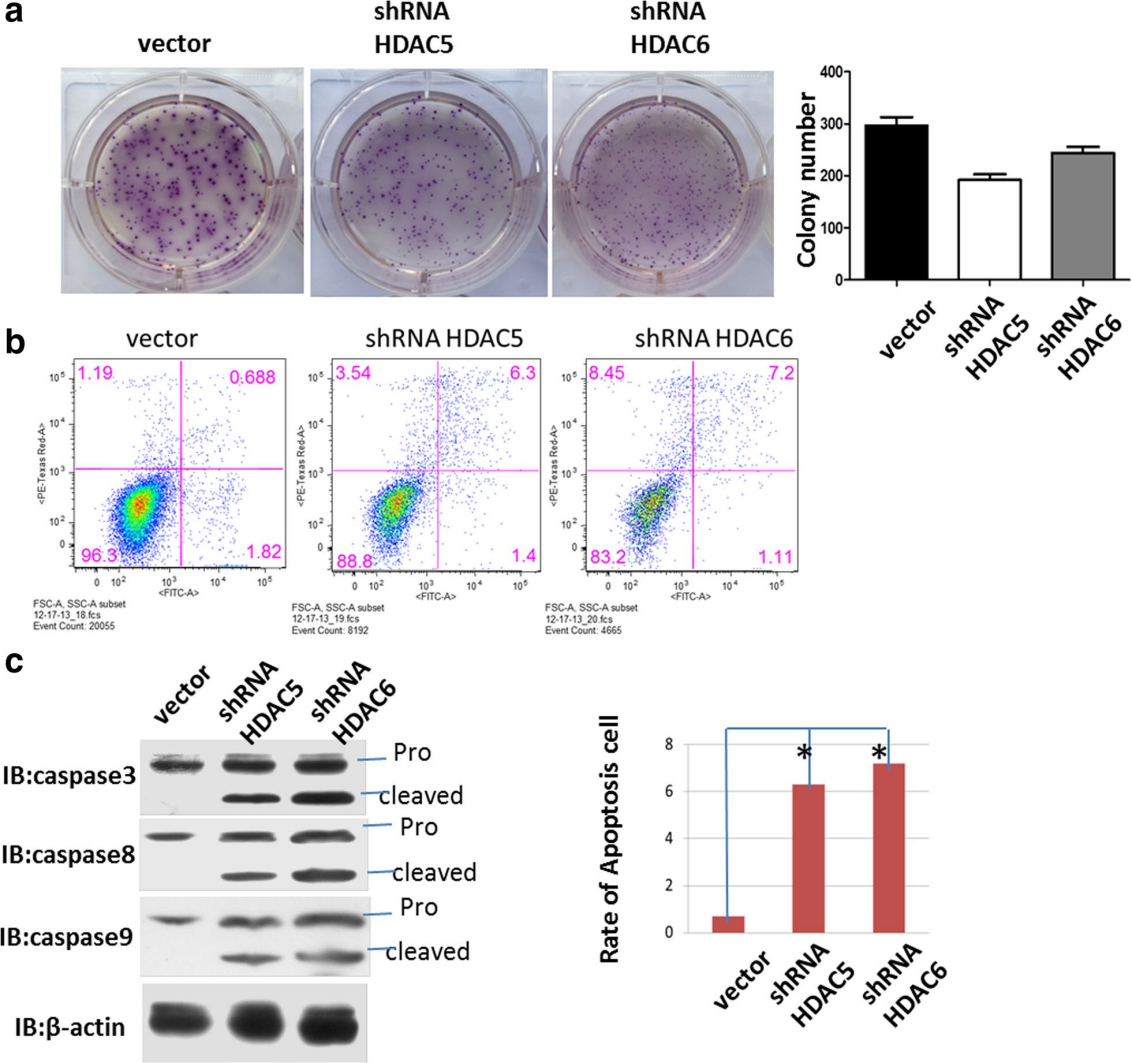
Knocking down HDAC5 or HDAC6 induced apoptosis in A375 cells. **a** Colony formation of Scr, HDAC5 shRNA, and HDAC6 shRNA stable cell lines. Knocking down HDAC5 or HDAC6 in A375 cells generated smaller colony of cells than the scr control, but presented close colony number. **b** and **c** Knocking down HDAC5 or HDAC6 induced apoptosis. Annexin V was used to stain the apoptotic cells, and the western blot of caspase 3, 8 and 9 also showed similar results: increased apoptotic cell numbers in stable cell lines of HDAC5 or HDAC6 knockdown. *p < 0.01, means large significance

Next, we assessed whether HDAC5 or HDAC6 knockdown induces apoptosis in A375 cells. Colony formation assay proved that reduced expression of HDAC5 or HDAC6 decreased cell growth and colonization. Also as shown in Fig. 3b, decreased expression of HDAC5 or HDAC6 promoted cell apoptosis, from 0.688 percent to 6.3 and 7.2 percent, respectively. The apoptosis pathway proteins caspase 3, 8 and 9 exhibited obvious cleaved bands, indicating that both HDAC5 and HDAC6 could modulate apoptosis by converging on the same pathway. We suspect there is a correlation between decreased colonization and increased apoptosis after HDAC5 or HDAC6 knockdown. Fan and Qin et al. [36, 37] found that inhibition of HDAC5 in HCC hep3B cells or inhibition of HDAC6 in Hela cells induces apoptosis. In Fig. 2c, after an initial growth, cells expressing the shRNA against HDAC6 seem to die (subsequently to day 5). For clarifying this, cytofluorimetric analysis for Annexin V/PI positivity was used to measure the percent of apoptotic and dead cells after Knocking down HDAC6 for 1, 3, 5 and 7 days (Additional file 3: Figure S3).

**Fig. 4:**
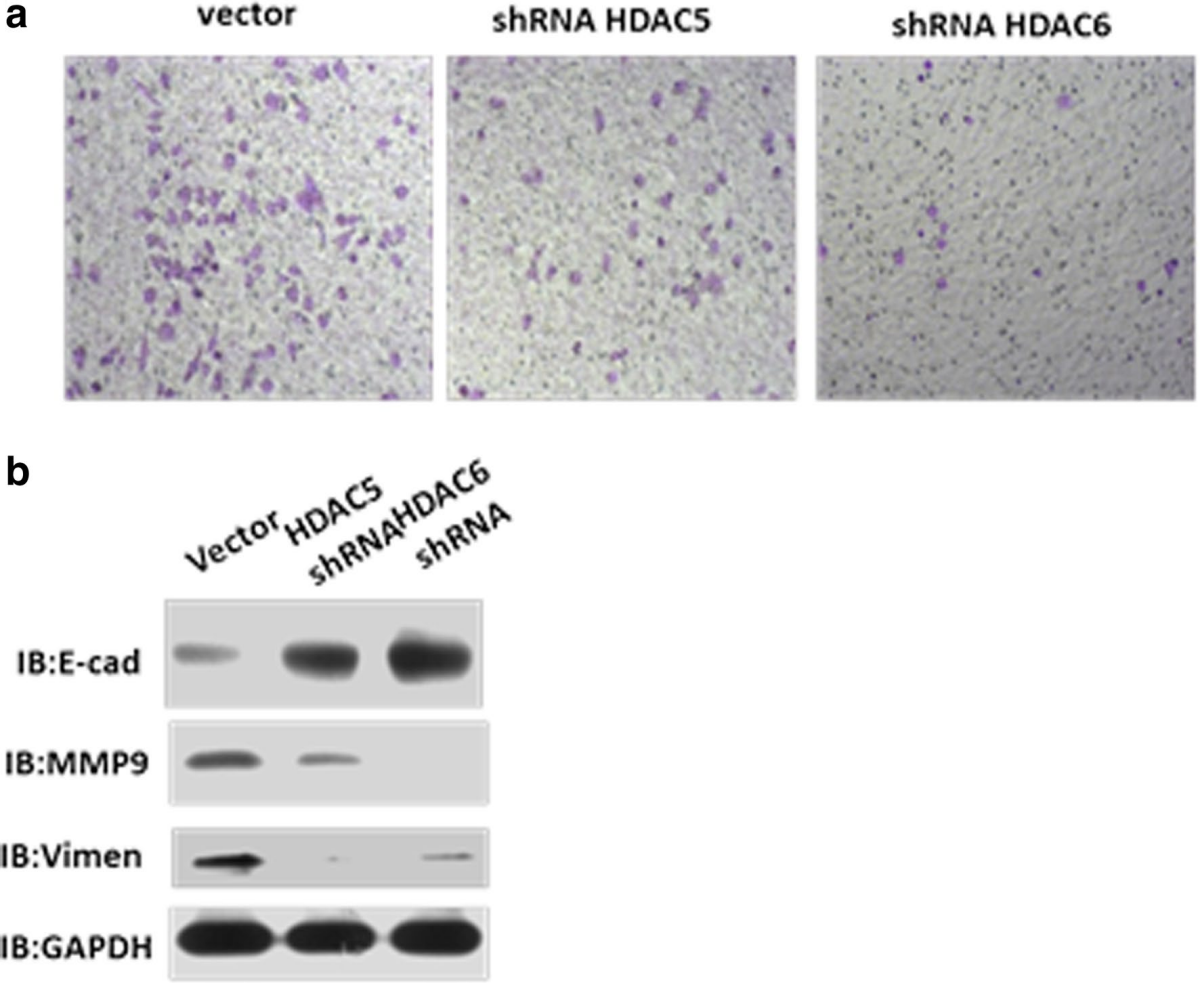
HDAC5 and HDAC6 influenced the metastasis of A375 cells. **a** Transwell assay to detect metastasis of A375 cells. Cell transwell assays were performed using FalconTM Cell Culture Inserts (BD353097) according to the manufacturer’s instructions. After 24–48 h of incubation, the remaining cells in the upper chamber were removed by cotton swabs. The cells on the lower surface of the membrane were fixed in 4 % paraformaldehyde and stained with 0.5 % crystal violet. Cells in at least three random microscopic fields (magnification x10) were counted and photographed. All experiments were performed in duplicate and repeated three times. **b** Western blot showing that knocking down HDAC5 or HDAC6 modulated metastasis through divergent pathways

We also assessed the metastasis ability in vitro through transwell assays. As shown in Fig. 4, both HDAC5 and HDAC6 could influence the metastasis of A375 cells by regulating MMP9 and vimentin, both markers for the metastasis ability of cancer cells. Many clinical melanoma patients have BRAF V600 mutation, such as V600E or V600D which confers melanoma cells the capacity of metastasis. In order to clarify the correlation of HDAC5,6 with BRAF V600E or V600D mutation, we knocked down HDAC5 or HDAC6 in M257 cells (BRAF wild type), SK-MEL-28 and A2058 (BRAF V600E),WM266 (BRAF V600D). Generally, we got the results which were consistent with Fig. 4b, when knocking down of HDAC5 or HDAC6, the melanoma cells dramatically decreased the metastasis ability (Additional file 4: Figure S4), indicating HDAC5 and HDAC6 may function at the downstream of BRAF.

### Both HDAC5 and HDAC6 promote cell cycle of melanoma cells

**Fig. 5:**
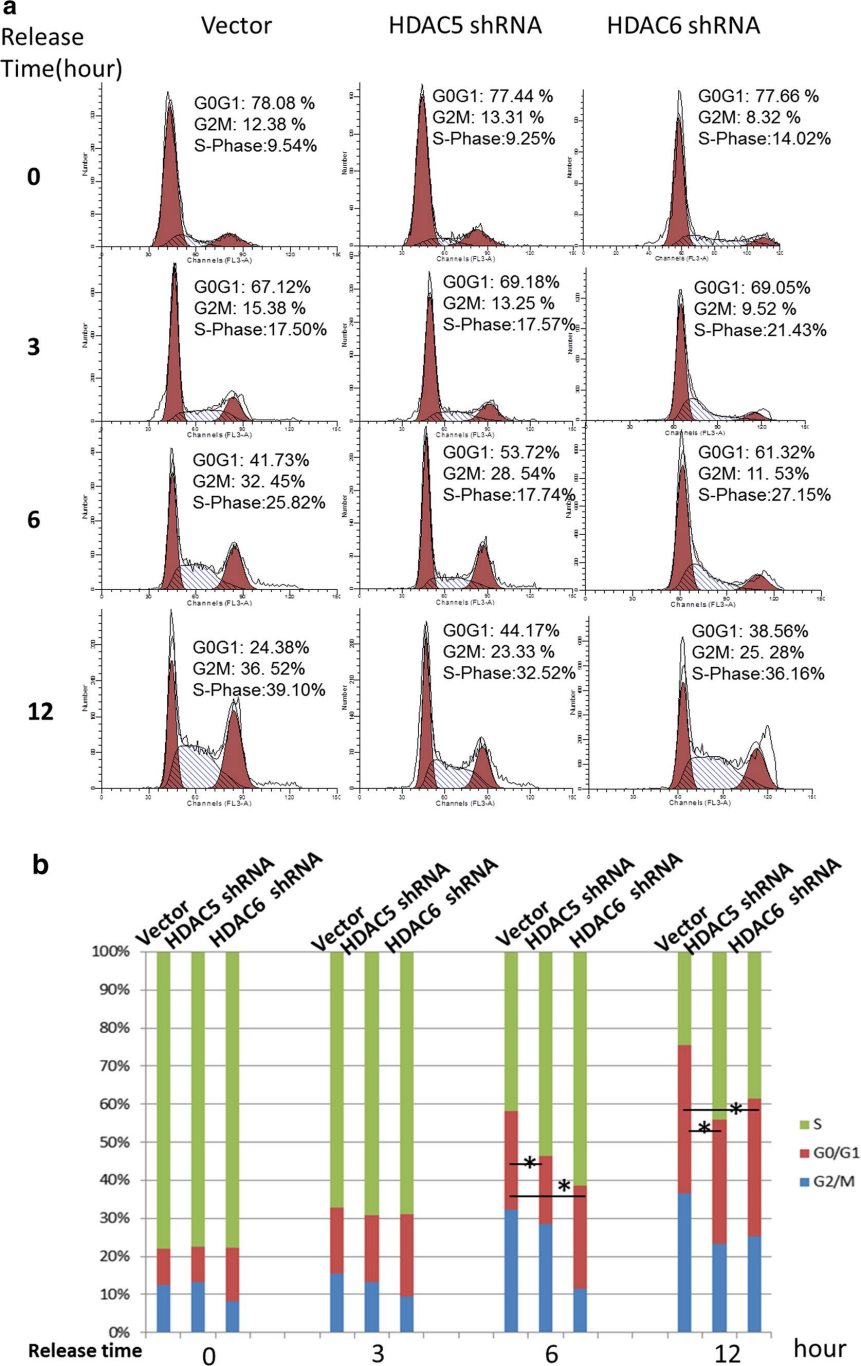
Knockdown of HDAC5 or HDAC6 induced cell cycle arrest. **a** and **b**, Cells were passaged in 12-well plates for 24 h. Serum was removed from the medium, and cells were starved with serum-free medium for 24 h. Then, the cells were cultured with fresh medium with serum at four time points: 0, 4, 8 and 16 h. After harvesting and washing with PBS, all of the A375 cells were fixed in ice-cold 70 % EtOH for more than 2 h at 4 °C, centrifuged at 2000 rpm for 5 min, then washed twice in PBS. The cells were incubated at 37 °C Rnase solution (1 mg/ml) for less than 30 min and then stained by propidium iodide (50 µg/ml) for cell cycle analysis with Flow Cytometers Galios (Beckman, USA) using a 488 nm excitation wavelength, gating doublets and clumps using pulse processing and collecting fluorescence above 620 nm. Data were extracted with Modfit software.*p < 0.05 was considered as significant difference

We also analyzed the cell cycle of the following three stable cell lines: scr, HDAC5 shRNA, and HDAC6 shRNA in A375 cells. As shown in Fig. 5, both HDAC5 and HDAC6 were required for normal cell cycle progression. Over 50 % Scr cells escaped from G0/G1 phase after releasing to fresh medium for 12 h while HDAC5 shRNA and HDAC6 shRNA groups were about 30 % and near 40 %, respectively. Our results demonstrate that knockdown of HDAC5 or HDAC6 arrested cell cycle in G0/G1 phase, indicating that HDAC5 and HDAC6 are required for regulating the expression of cell cycle-related genes and promote A375 cells progressing to S phase. Also, we analyzed CDKN1A expression in HDAC5 shRNA cells and the data in Additional file 5: Figure S5 showed that knocking down HDAC5 promoted CDKN1A expression, indicating inhibition of HDAC5 released the repression of MEF2-dependent transcription [38].

### Effect of HDAC5 or HDAC6 knockdown on tumor growth

**Fig. 6:**
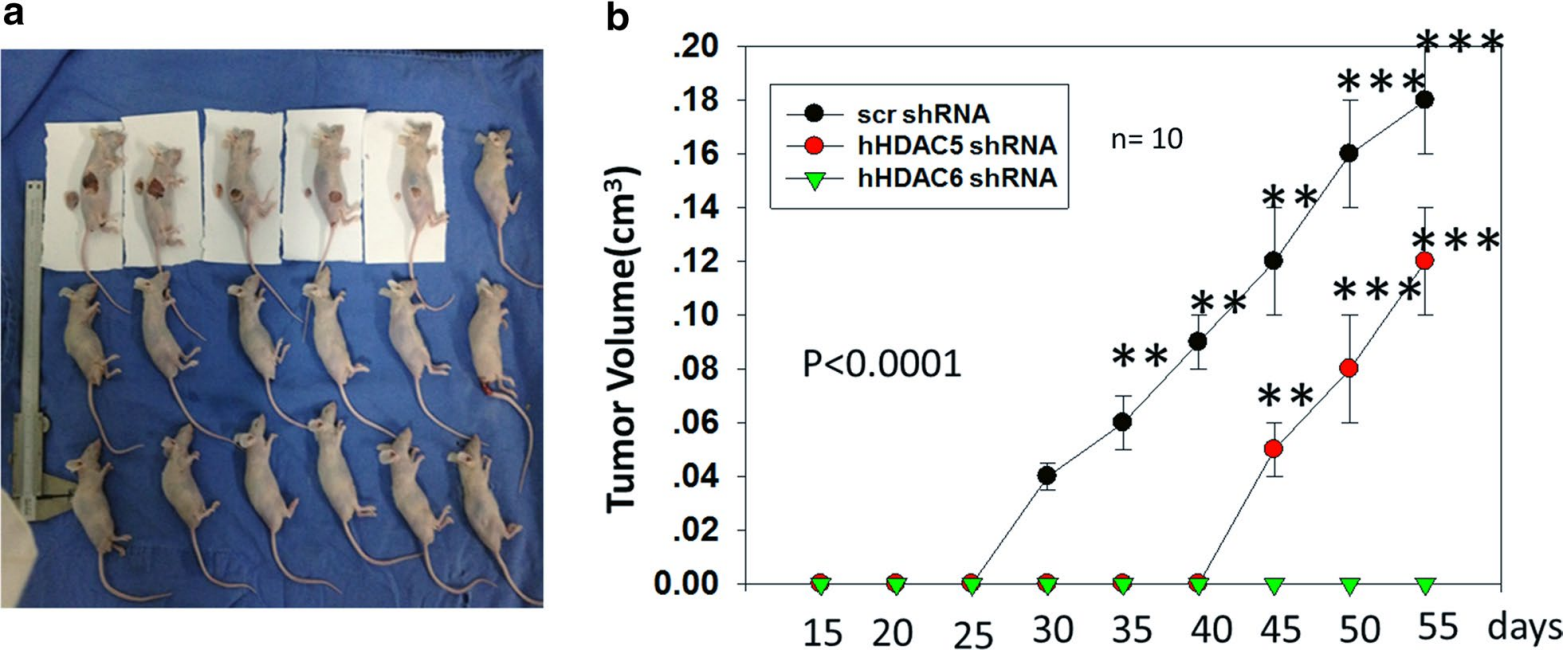
Both HDAC5 and HDAC6 were required for tumor growth. **a** Knocking down HDAC5 or HDAC6 inhibited the tumor growth of the subcutaneously transplanted A375 cells (2 x 10^6^). Experimental groups: A375 Scr; A375 HDAC5 shRNA; A375 HDAC6 shRNA, each performed in four nude mice. **b** Mean tumor volume was measured at the indicated number of days after A375 cells (2 x 10^6^) were implanted into the armpit of the nude mice. *p < 0.05, **p < 0.001, ***p < 0.0001. Bars, s.d

We further investigated the effects of HDAC5 or HDAC6 knockdown on A375 cells tumor growth when the cells were transplanted subcutaneously. As shown in Fig. 6, HDAC5 or HDAC6 knockdown dramatically inhibited tumor growth, especially HDAC6 knockdown, indicating that HDAC6 may have a more serious influence on cancer cell growth. As shown in Fig. 6a, HDAC5 and HDAC6 knockdown induced a 25 and 98 % reduction in the tumor volume of A375 cells, respectively. We analyzed the mice survival rate after injecting A375 stable cells into null mice. We found consistent results with tumor growth after injecting scramble A375 cells for 3 weeks; the null mice (n = 10) group lost four mice, whereas the HDAC5 or HDAC6 knockdown groups had no mice dead.

### HDAC5 and HDAC6 were up-regulated in clinical melanoma tissues

After lung and breast cancer, melanoma is one of the most common underlying diagnoses in patients with cerebral metastases [39]. HDAC inhibitors, such as hexamethylene bisacetamide (HMBA) and MS275, have been reported to suppress the progression of melanoma in clinical or in vitro experiments. Additionally, HDAC inhibitors, such as TSA and SB, can induce apoptotic cell death in a number of tumor cell types, including melanoma [40, 41].

**Fig. 7:**
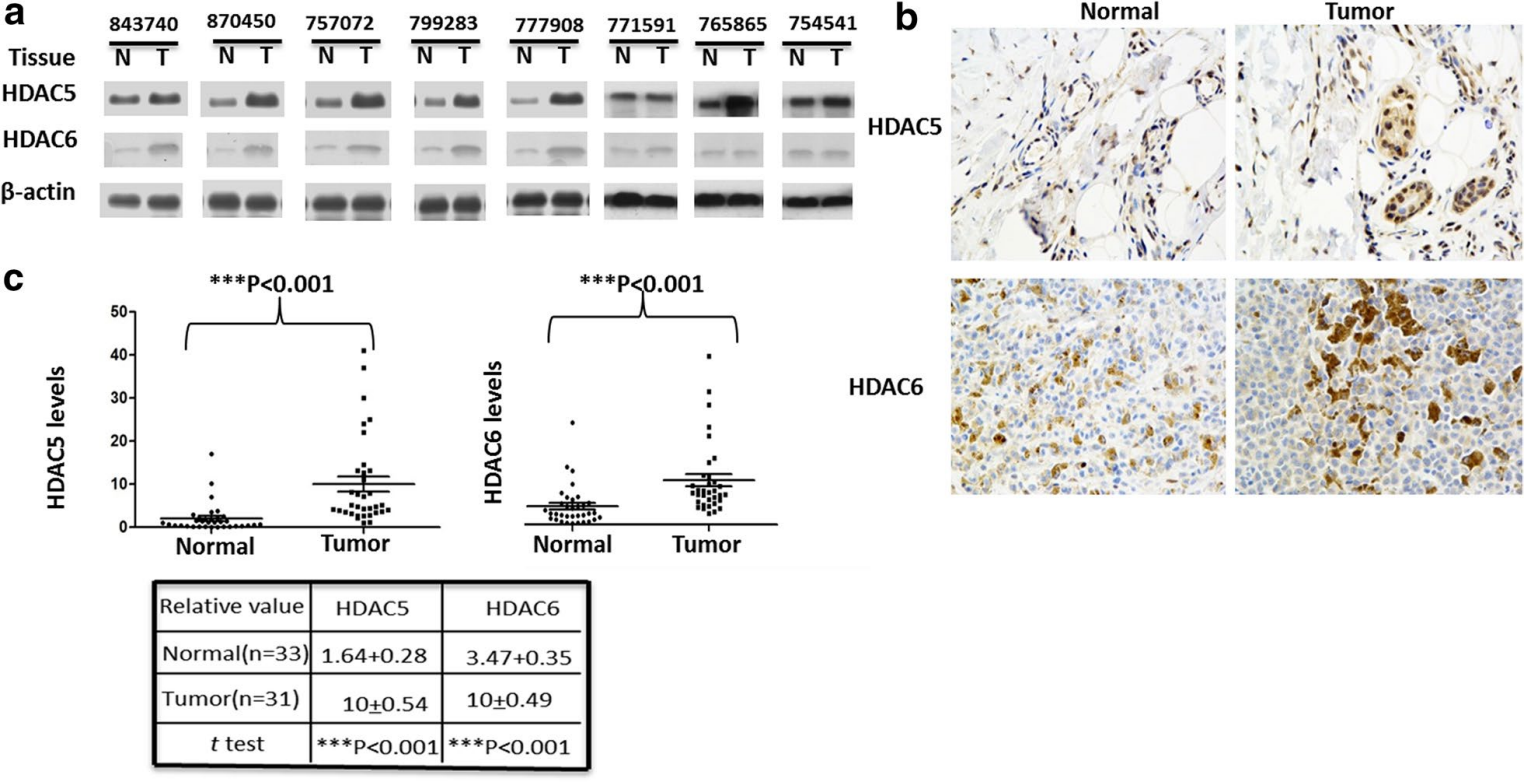
HDAC5 and HDAC6 were up-regulated in melanoma tissues. **a** Total HDAC5 or HDAC6 protein levels were are increased in melanoma tissues compared to adjacent tissues. The protein levels of HDAC5 and HDAC6 in 8 pairs of melanoma and adjacent normal tissues were analyzed by western blotting. **b** and **c** Immunohistochemical stainings of HDAC5 and HDAC6 proteins in tumor and adjacent normal tissues. One example is shown in **b**, and the statistical analysis of all samples is shown in **c**. Scale bars are 50 mm. The intensities of the total HDAC5 (*upper panel*) and HDAC6 (*down lower panel*) proteins were quantified using the Motic Images Advanced software, followed by statistical analysis. A total of 33 tumor tissues and 31 adjacent normal skin tissues were analyzed. The mean value of multiple samples and standard deviation are presented. ***p <0.001

HDAC5 or HDAC6 knockdown in A375 cells induced apoptosis, arrest of cell cycle and tumor growth in nude mice. Furthermore, we collected 64 primary human melanoma samples, including 31 pairs that had surrounding normal skin tissues. We first carried out a western blot analysis for a panel of 8 pairs of primary melanoma (T) and their adjacent normal tissues (N), for which sufficient amounts of proteins were obtained. This analysis revealed that compared with normal skin tissues, 6 pairs showed a significant increase of the steady-state levels of total HDAC5 protein, whereas 5 pairs showed increased HDAC6 (Fig. 7a).

However, western blots did not demonstrate the specific location of HDAC5 or HDAC6 in melanoma tissues. For clarifying the sublocations of HDAC5 and HDAC6 in melanoma tissues, we then performed immunohistochemistry in 10 clinical melanoma samples, including 46 samples that had the adjacent normal melanoma tissues. In most samples, the levels of total HDAC5 and HDAC6 were higher in melanoma tissues than normal skin tissues (Fig. 7b). Analyses of the quantified images indicated that the differences between tumor and normal tissues in total HDAC5 or HDAC6 levels protein levels (P < 0.0001) were all highly significant when comparing the 33 tumor samples with the 31 normal skin samples (Fig. 7c).

## Discussion

HDAC inhibitors have contributed extensively to the prevention of melanoma cell proliferation and metastasis. Multiple small molecules have emerged as inhibitors of HDACs, with consequences for chromosome remodeling, cell cycle arrest and selective toxicity in cultured melanoma cells compared with normal melanocytes [6, 42]. Increasing evidence indicates that HDAC inhibitors have multiple effects on tumor cells, including induction of apoptosis, arrest of cell cycle to slowdown cell proliferation, and induction of differentiation or autophagy, etc. [43].

HDAC inhibitors have made great progress as anti-cancer agents, including the HDAC inhibitor panobinostat, developed by Novartis; Zolinza, an HDAC inhibitor developed by MERCK and on the market since 2006; Istodax, an HDAC inhibitor developed by Celgene and FDA approved in 2010; and other new drugs that are in preliminary trials, most of which are new HDAC inhibitors. Recently, a second generation of reportedly available HDACis have been tested in the clinic including the class I—specific agents CHR-3966 [14], chidamide (CS055/HBI-8000) [15], class I— and class II—specific AR-42 [16], and hydroxamides quisinostat (JNJ-26481585) [17] and abexinostat (PCI-24781) [18]. However, despite the large number of positive reports, the limitations of HDAC inhibitors are also increasingly clear. For example, we do not know which one specific HDAC must be inhibited or whether HDACs inhibition would present a collective effect. Additionally, the concrete substrate groups of HDACs and mechanisms of action are unknown.

Here, we showed the expression levels of all 11 HDACs in multiple melanoma cells comparing with normal skin cells and found that HDAC5 and HDAC6 have lower expression levels in normal tissues but much higher expression in A375 and A2058 cells. Thus, we suggest that HDAC5 and HDAC6 may contribute to the occurrence of melanoma.

HDACs have broad range of substrates, including some transcriptional factors such as p53, STAT3, FoxO1, etc. According to the literature, HDAC5 has a wide range of substrates, including RunX2 and PKM2.HDAC4 and HDAC5 usually forms a complex to deacetylate substrates. For example, HDAC4 and HDAC5 deacetylate Runx2, allowing the protein to undergo Smurf-mediated degradation [44]. HDAC5 has been shown to promote PKM2 interaction with FBP (fructose 1,6-bisphosphate) through deacetylation of PKM2 K433, which is a key step to activate PKM2 kinase activity [45]. In most cases, HDAC6 is located in the cytoplasm and tends to acetylate microtubules, which exist in multiple functional systems. For example, macrophages challenged by bacterial lipopolysaccharides (LPS) undergo extensive microtubule acetylation by HDAC6, thereby reversing the acetylation process [46].

Knocking down HDAC5 or HDAC6 in A375 cell line suppressed its proliferation and induced apoptosis. Using mouse models with subcutaneous tumor xenografts grown from implanted A375 cells, we observed smaller tumors from A375 cells expressing ShRNA against HDAC5 or HDAC6. Interestingly, although both HDAC5 and HDAC6 contribute to maintain cell proliferation in A375 cells, these two HDACs belong to different HDAC families; HDAC5 belongs to HDAC class IIa, whereas HDAC6 belongs to HDAC class IIb. Typically, HDAC5 is a nuclear protein, but it can also translocate from the nucleus to cytoplasm during injury or stress on peripheral neurons and can enhance histone acetylation to activate a pro-regenerative gene-expression program [19]. By contrast, HDAC6 is a cytoplasmic protein with two repeat catalytic domains located in the N terminus, whereas most HDACs have only one catalytic domain in the C terminus [47]. HDAC6 has a zinc finger structure in the C terminus, which is different from other HDACs, and the zinc finger structure has ubiquitin ligase activity and can bind with ubiquitin. Therefore, HDAC6 can be degraded via the ubiquitin-dependent pathway [48, 49]. Therefore, HDAC5 and HDAC6 may have different and a broad range of substrates affecting multiple signaling pathways.

Furthermore, there is communication between the families of HDACs and SIRTs. For example, Mihaylova et al. reported that class IIa HDAC4 and HDAC5 could recruit HDAC3, which led to the acute transcriptional induction of gluconeogenic enzymes genes via deacetylation and activation of FOXO family transcription factors [50]. Yang et al. [51] reported that the deacetylases HDAC6 and SIRT2 co-regulated the acetylation state of K-RAS in cancer cells. Stefan et al. showed that class I and IIa HDACs have different expression levels in constitutively SOST-expressing UMR106 osteocytic cells and exert opposite effects on sclerostin gene regulation [52].

HDAC inhibitors can also act in concert with other oncogenic targets to achieve better inhibition of tumor cell growth. For example, Lai et al. [53] synchronously adopted the inhibitors of HDACs and oncogenic BRAF and effectively killed melanoma cells. Additionally, Sun et al. [54] showed that HDAC5 interacts with N-myc for blocking neuroblastoma cell differentiation.

Knockdown of HDAC5 or HDAC6 can cause apoptosis and cell cycle arrest, meanwhile, suggesting that there is no functional redundancy in A375 melanoma cells between HDAC5 and HDAC6. Our results provide the basic theoretical foundation for the combined applications of HDAC inhibitors to treat cancer and simultaneously inspire researchers further to investigate the HDAC targeting proteins which are formed that form a complex networks.

## Conclusions

This study provided evidence for the first time that melanoma specifically overexpressed HDAC5 and HDAC6. We presented both HDAC5 and HDAC6 as tumor promoters in melanoma proliferation and metastasis through different signaling pathway, and shaded some light on the potential mechanisms. Our results provided the basic theoretical foundation for the combined application of HDAC inhibitors to treat cancer and simultaneously inspired researchers to further investigate the HDACs targeting proteins that form complex networks. We believe that both HDAC5 and HDAC6 could be good diagnostic and therapeutic targets for controlling melanoma.

## Additional files

10.1186/s12967-015-0753-0 HDAC5 and HDAC6 were overexpressed in melanoma cells, and qRT-PCR was used to identify the expression level of all the HDACs except sirtuins in A375 cells, A2058 cells and normal skin cells: HaCat cells. All of HDACs expression levels were firstly normalized to GAPDH and then ratio to HaCaT cells.
10.1186/s12967-015-0753-0 Screening for an efficient shRNA for HDAC5 or HDAC6 knockdown. The seq used for RNA interference are listed in Materials and Methods. HDAC5 (or HADC6) shRNA vectors were transiently transfected in HEK293T cells, and the cells were collected 36 h later, washed twice with ice cold PBS, and centrifuged at 1000 rpm for 5 min. Then, 1 × SDS loading buffer was added and boiled for 10 min; then 10 μl of samples was loaded for SDS-PAGE. β-actin was used as an internal control.
10.1186/s12967-015-0753-0 Knocking down HDAC6 induced apoptosis with time course. Annexin V was used to stain the apoptotic cells and PI was used to stain the dead cells. After constructing knocking down HDAC6 stable cells, we continued to culture these cells in RPMI1640 medium and collected cells with a time course: 1, 3, 5 and 7 days.
10.1186/s12967-015-0753-0 The effect of HDAC5 and HDAC6 on metastasis didn’t depend on BRAF V600 mutation. a knocking down HDAC5 or HDAC6 in multiple BRAF V600 mutation cells. b Transwell assay to detect metastasis of A375 cells. Cell transwell assays were performed using FalconTM Cell Culture Inserts (BD353097) according to the manufacturer’s instructions. After 24–48 h of incubation, the remaining cells in the upper chamber were removed by cotton swabs. The cells on the lower surface of the membrane were fixed in 4 % paraformaldehyde and stained with 0.5 % crystal violet. Cells in at least 3 random microscopic fields (magnification ×10) were counted and photographed. All experiments were performed in duplicate and repeated 3 times.
10.1186/s12967-015-0753-0 knocking down HDAC5 elevated CDKN1A expression. GAPDH was used as normalized control.
