# Supplementary figures and images for: Both HDAC5 and HDAC6 are required for the proliferation and metastasis of melanoma cells
Source: J Transl Med. 2016 Jan 8;14:7. doi: 10.1186/s12967-015-0753-0 (PMC4706654; doi:10.1186/s12967-015-0753-0)

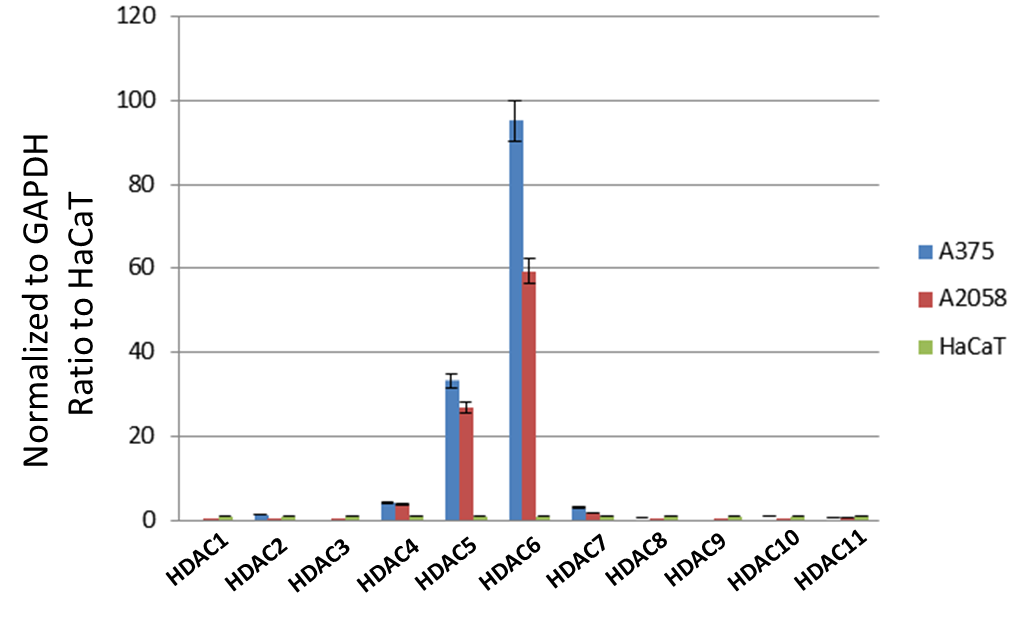

Supplement: Supplementary file 1 — 10.1186/s12967-015-0753-0 HDAC5 and HDAC6 were overexpressed in melanoma cells, and qRT-PCR was used to identify the expression level of all the HDACs except sirtuins in A375 cells, A2058 cells and normal skin cells: HaCat cells. All of HDACs expression levels were firstly normalized to GAPDH and then ratio to HaCaT cells. [file 12967_2015_753_MOESM1_ESM.tif]

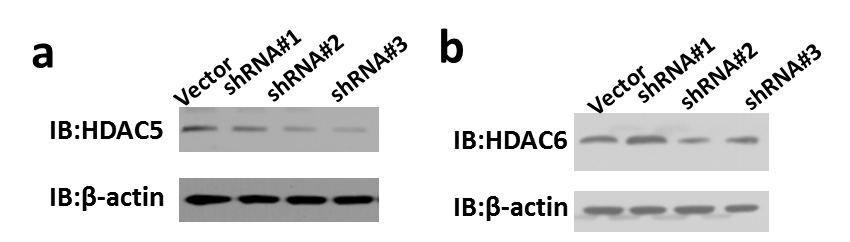

Supplement: Supplementary file 2 — 10.1186/s12967-015-0753-0 Screening for an efficient shRNA for HDAC5 or HDAC6 knockdown. The seq used for RNA interference are listed in Materials and Methods. HDAC5 (or HADC6) shRNA vectors were transiently transfected in HEK293T cells, and the cells were collected 36 h later, washed twice with ice cold PBS, and centrifuged at 1000 rpm for 5 min. Then, 1 × SDS loading buffer was added and boiled for 10 min; then 10 μl of samples was loaded for SDS-PAGE. β-actin was used as an internal control. [file 12967_2015_753_MOESM2_ESM.tif]

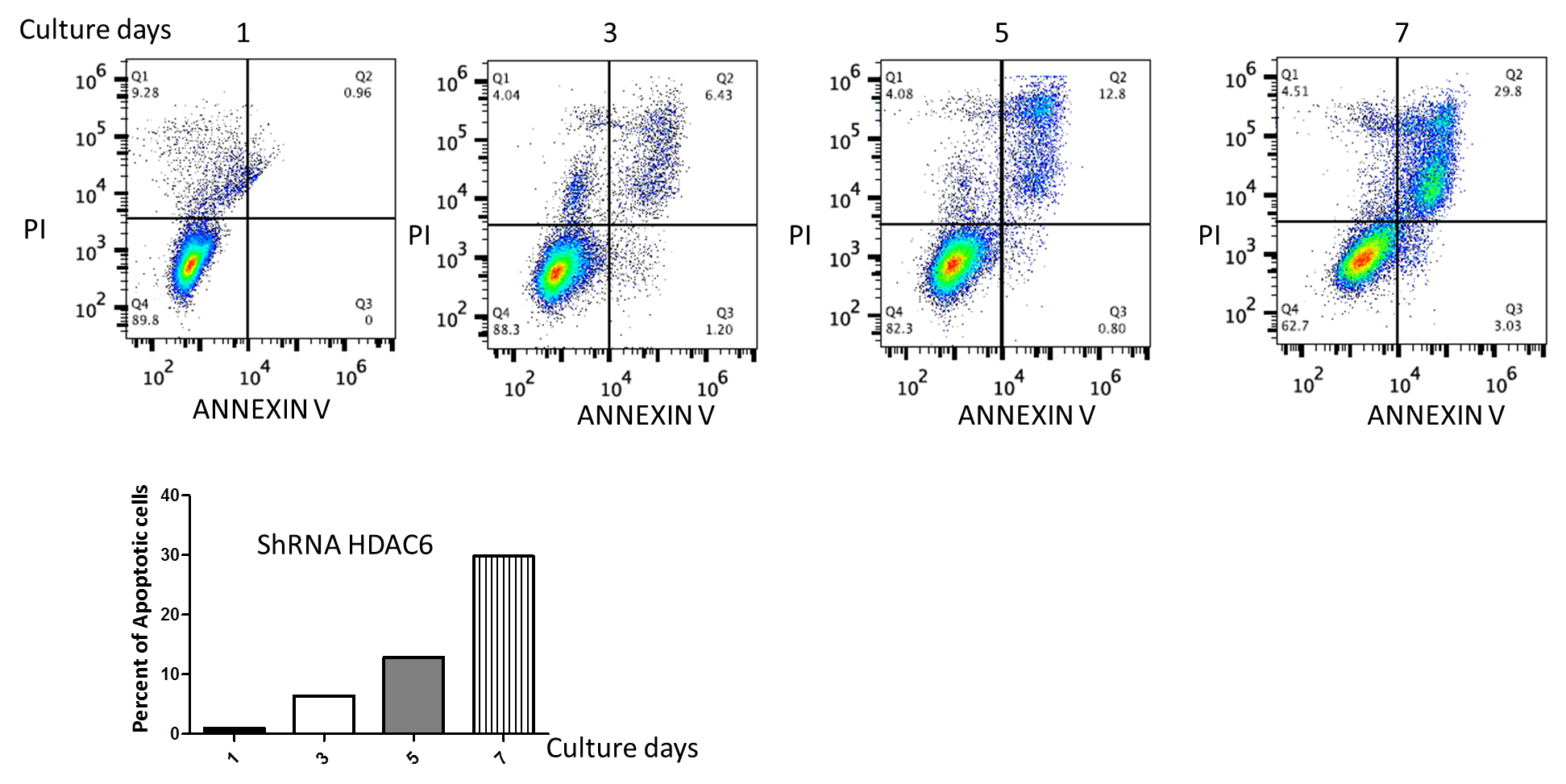

Supplement: Supplementary file 3 — 10.1186/s12967-015-0753-0 Knocking down HDAC6 induced apoptosis with time course. Annexin V was used to stain the apoptotic cells and PI was used to stain the dead cells. After constructing knocking down HDAC6 stable cells, we continued to culture these cells in RPMI1640 medium and collected cells with a time course: 1, 3, 5 and 7 days. [file 12967_2015_753_MOESM3_ESM.tif]

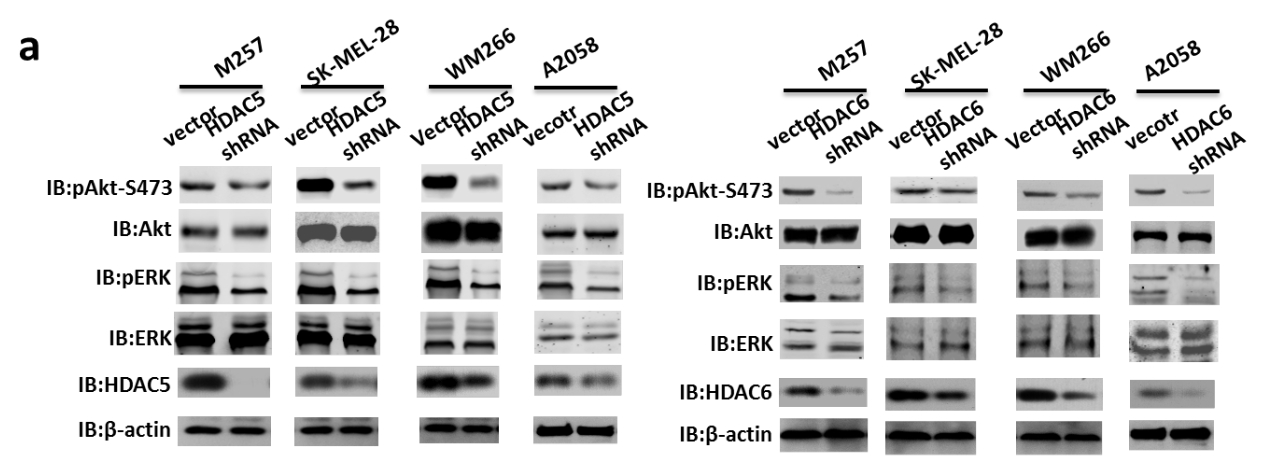


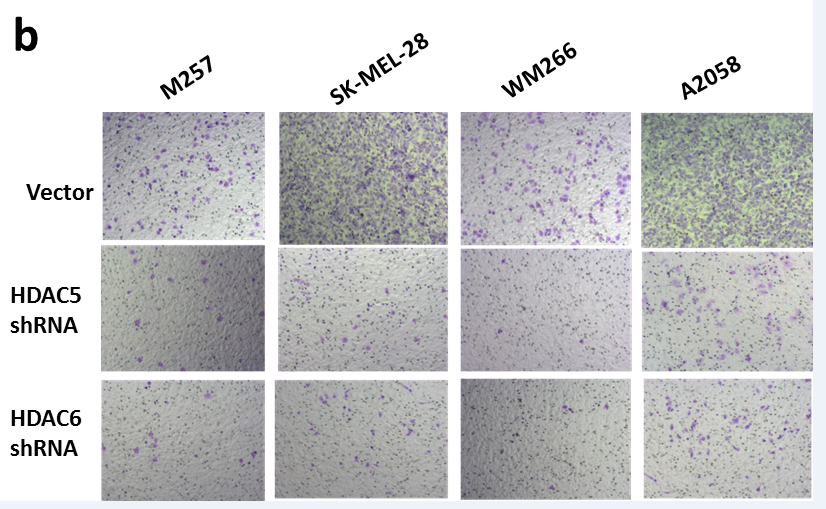

Supplement: Supplementary file 4 — 10.1186/s12967-015-0753-0 The effect of HDAC5 and HDAC6 on metastasis didn’t depend on BRAF V600 mutation. a knocking down HDAC5 or HDAC6 in multiple BRAF V600 mutation cells. b Transwell assay to detect metastasis of A375 cells. Cell transwell assays were performed using FalconTM Cell Culture Inserts (BD353097) according to the manufacturer’s instructions. After 24–48 h of incubation, the remaining cells in the upper chamber were removed by cotton swabs. The cells on the lower surface of the membrane were fixed in 4 % paraformaldehyde and stained with 0.5 % crystal violet. Cells in at least 3 random microscopic fields (magnification ×10) were counted and photographed. All experiments were performed in duplicate and repeated 3 times. [file 12967_2015_753_MOESM4_ESM.docx]

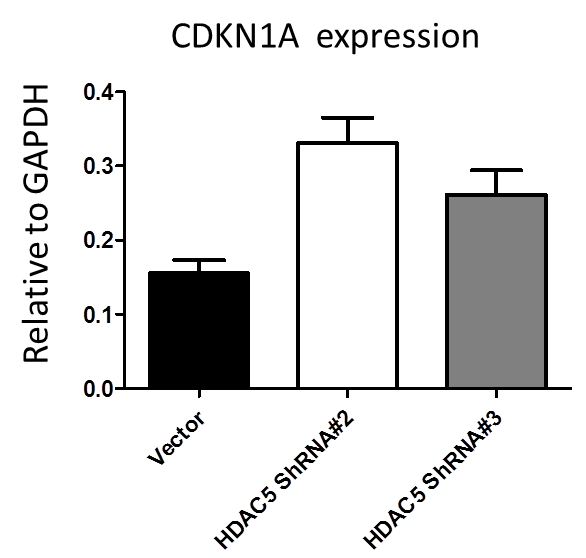

Supplement: Supplementary file 5 — 10.1186/s12967-015-0753-0 knocking down HDAC5 elevated CDKN1A expression. GAPDH was used as normalized control. [file 12967_2015_753_MOESM5_ESM.tif]
